# Miscellanies

**Published:** 1834-07-01

**Authors:** 


### MISCELLANIES.

ST. PANCRAS MEDICAL ASSOCIATION, FOR PROMOTING AND CARRYING INTO EFFECT A SELF-SUPPORTING CHARITABLE AND PAROCHIAL DISPENSARY,

"*By the adoption of which our philanthropy may be reduced to a system, our best class of poor prevented from retrograding into pauperism, and our necessitous and legitimate paupers, separated from the comparatively improvident and vicious.*"

PRINCIPLE AND OBJECT OF THE INSTITUTION.

1st, To place within the reach of the industrious artizan of small income the means of obtaining medical aid, without subjecting him to the necessity of seeking humiliating charity, or destroying his honest pride, by exposing him to the demoralising influence of the poor laws.

2d, To afford gratuitous relief to persons of real desert, who may be unable to procure by their own efforts the advantages above described.

The first-mentioned or FREE CLASS of patients, may obtain for themselves surgical and medical attendance by assuring with the small sum of sixpence per month, which is not equal to the amount frequently wasted by them on nostrums and self-prescriptions. In virtue of this premium they may demand and obtain the most efficient professional assistance and medicines, and to their demands, the number of surgeons ensures immediate attention; thus will be saved the time and labour often fruitlessly, and always painfully bestowed on seeking the signature of a governor of an honorary dispensary; the unfortunate will be spared the bitter degradation of asking charity, for which will be substituted the honorable satisfaction of claiming assistance as a right, and obtaining it immediately.

The second object of this institution will be attained by the aid of the charitable and humane, who are entreated to be discriminating in the bestowal of their kindness, and thus insure the legitimate application of their bounty to persons whose destitution unhappily precludes them from the benefits which the industry and self-respect of the First Class (the free members) procure for themselves.

As the sum which will entitle the donor to have a patient constantly under medical care, is only half the amount invariably demanded by honorary dispensaries, those who are anxious to give the most extensive effect to their humunity, will see the advantage of supporting this Institution. The means by which this superiority is obtained over honorary dispensaries are the following :—

The surgeons constituting this Association will dispense their own prescriptions, thus saving the cost of keeping up a dispensary-house and appendages, and salary of dispensing apothecary, a charge equal to at least a third of the whole income of such institutions ; in other words, of every guinea subscribed by the humane and excellent supporters of these charities, less than 14s. are appropriated to the literal benefit of the poor. This obvious advantage will enable the St. Pancras Medical Association to allow subscribers of one guinea annually to have two patients constantly on the books, and subscribers of half a guinea, one. For proportionate increase of subscription, corresponding privileges are allowed.

| *Surgeons.* | *Days of Attendance.* |
|---|---|
| Mr. Malim, 14, Regent square<br>Mr. Cumming, 28, Tavistock-place<br>Mr. Robinson, Seymour-street | Monday and Thursday. |
| Mr. Fraser, 5, Clarendon Square, Somers Town<br>Mr. Milbanke, Judd St.<br>Mr. Cumming, 28, Tavistock-place | Tuesday and Friday. |
| Mr. Rhodes, 54, Judd St.<br>Mr. Winter, Tavistock-place<br>Mr. Walford, 39, Speldhurst Street | Wednesday and Saturday. |

Application to be made to any of the surgeons of the day from 10 till 12 o'clock. The surgeon to whom application is made, will continue to attend to the case till its termination.

*N.B. Any of the surgeons may be consulted till* 11 *o'clock on Sunday.*

EXTRACTS FROM THE LAWS AND REGULATIONS.

18. Surgeons shall prescribe and furnish medicines at their own houses unless the patient be too ill to go out ; he shall then be visited by his surgeon at his (the patient's) own house.

19. The free members shall consist of working men, their wives, and children, *and of any other persons who may be unable to provide themselves with medical aid and drugs in the usual manner ;* the terms of subscription to be sixpence per month from each member above twelve years of age, and threepence per month from each member under that age.

20. Persons wishing to become free members, must send, or have sent for them, their names, age, and places of abode to any two members of the Association, who shall immediately present the same to the treasurer, and report it to the next committee meeting : any one of the surgeons failing to do this, shall be fined one shilling, to go into the general fund.

21. No free member shall be entitled to relief unless all arrears be paid up ; every free member in arrear one month shall be find 1*d.*, if two months 2*d.*, if three months, 3*d.*; and if a longer period, shall forfeit all benefit derivable from the Association. Each member shall at the time of his entering as a free member sign a paper agreeing to this law.

22. Poor married women, *who are free members,* may be attended in their confinement by any one of the surgeons they may please to select, on paying ten shillings as a fee, with the treasurer two months previous to their confinement.

23. The ten shillings so deposited to be paid by the treasurer to the surgeon who has attended the case, at the next quarterly general meeting succeeding the attendance.

24. All free members shall belong to the Institution for at least four months before they shall be entitled to the benefits thereof, but any person may become a free member by paying four months' subscription in advance, unless they apply for admission during sickness, then they shall pay twelve months subscription in advance.

25. Donors of ten shillings and sixpence annually, shall be entitled to have one patient always on the sick list; donors of one guinea annually, shall be entitled to have always two patients on the sick list; and for every additional half-guinea, they shall have the privilege in favour of an additional patient.

---

We have given insertion to the foregoing plan of a self-supporting Dispensary, and it will hardly be supposed that we can have the least wish to impede the operation of any measure calculated to improve the condition of the poor—instil into their minds feelings of independence—or induce the rich to contribute to the support of the indigent. We confess, however, that we entertain some fears that evils will accompany a scheme like the present one. The principle is, as nearly as possible, that of an insurance company, where a small sum is annually paid, to receive back, in case of necessity, (SICKNESS being placed instead of DEATH,) a valuable equivalent. This principle, in general, is praiseworthy; but whether it will apply in the particular instance of medical assistance, is doubtful. In the first place, it looks very like a trading speculation. The medical practitioners offer medicines and attendance to an individual for sixpence per month, taking the chance that the individual may not be ill at all, against that of sickness. There is something INFRA DIG. in this. If the object be mere charity, let the good deed be done gratuitously—if a pecuniary gain be the object, the speculation is objectionable. We acknowledge that the principle of holding out encouragement to the poor to contribute something, while in health, for what may be termed "a sore foot," is excellent; but the clause in the 19th article, which we have underlined, will enable great numbers who are perfectly able to employ the medical practitioner at a reasonable remuneration, to take advantage of this Institution, and procure physic and skill for six shillings per annum!!* We know that, even as it is, great numbers of the class attend as out-patients at hospitals, to save expenses. In the mode here proposed, they can do the thing still more comfortably for themselves, inasmuch as they can demand the *quid pro quo*, without subjecting themselves to the humiliation of seeking eleemosynary aid at a public institution. We submit these reflections to the consideration of our brethren, having no object in view but the respectability of the profession, and the benefit of society at large.

---

### SPINAL DEFORMITIES—MR. UNDERWOOD.

*Resolutions.*

1st. That this Meeting, being deeply convinced that an immense amount of human suffering and inconvenience originates in the evils connected with, and consequent upon, spinal and limb deformities, do determine to use all their exertions in promoting the success of the charity established by a few benevolent individuals, under the name of *Harrison's Infirmary*.

2d. That the printed report and the copy of laws be approved of by this Meeting, as calculated to render efficient the exertions of the benevolent, in affording relief to the distressed deformed.

3d. That as the funds of the charity are at present insufficient for the accommodation of patients of both sexes, that females only shall in the first instance be received, and that upon the weekly payment of One Guinea, besides as many poor patients as the funds are able to maintain, in a separate part of the building.

4th. That the warmest thanks of this Meeting be given to Her Royal High-

---

* Who is to judge whether these "other persons" be able or not to provide themselves with "medical aid and drugs," in the "usual manner?"

ness the Duchess of Kent, for having been graciously pleased to become the Patroness of the Infirmary, and also for her promise of annual support.

5th. That the thanks of this Meeting be given to the President, John Underwood, Esq. for his exertions in the cause of this Charity.

6th. That the thanks of this Meeting be given to Dr. Harrison, for his noble donation of One Thousand Pounds.

7th. That the thanks of this Meeting be given to Drs. Harrison and Serney, and Messrs. Thornber and Hoyland, Esqrs. who have offered their gratuitous medical and surgical aid to the Charity.

8th. That Subscriptions be received by Messrs. Coutts and Co., John Underwood, Esq., and C. W. Hoyland, Esq.

N.B. All applications for the admission of patients to be made to the honorary Secretary, C. W. Hoyland, Esq. 15, Blandford Street, Portman Square.

---

### The Concours.

This subject has been warmly discussed by some of our contemporaries; but we have not yet adverted to it ourselves. It does not seem to have worked well, or at least satisfactorily, in a neighbouring country; and we doubt whether it will ever work at all on this side of the Channel. We think that public trials of strength for appointments to medical institutions should be based on public reputation, and not upon the facilities of answering questions in the minute or recondite branches of medical science. Such a tentamen as this last is the proper one for the student, who has concluded his course of education, and seeks a diploma as a proof of his qualifications to practise his profession. But when a practitioner becomes a candidate for a public institution, his claims should rest on his past labours, and his merits should be weighed by his professional brethren *alone*, because they *alone* are capable of appreciating them. On this account we could wish, though we do not expect, a fair and open election of medical officers by the general constituency of all qualified practitioners. In all elections there will be corruption, or corrupt influence (which is nearly the same thing), but the broader the constituency, the less opportunity would there be for nepotism and favouritism. The present mode of electing medical officers, through the medium of governors, who can be no judges of the comparative merits of the candidates, is deplorably bad. The public institutions themselves, as well as the public at large, and the medical profession, have often suffered by such a system of election, and will continue to suffer, till a more rational and a fairer mode be adopted. We reiterate our conviction, that the best concours would be the suffrages of the profession at large by a public election.

---

### Medical Reform.

Since the Parliamentary Committee on medical inquiry commenced its sittings, medical discussions have almost ceased, and that, too, from intensity of interest, rather than from diminution of excitement. Whoever has had the good or the bad fortune to be in a fleet or an army, on the point of coming into destructive conflict with the enemy, will remember the awful stillness, the death-like silence, that reigns on both sides of the hostile line. Something of this kind now obtains. The "collision" between the upper and the lower house of physic may be said to have taken place, and both parties stand in breathless expectation or anticipation of the result. Of the *final* result, we have little doubt. As, in general politics, the sun of high tory principles has set for ever, so, in medicine, the monopoly of rank and riches, without talent and industry, has seen its acmé, and will experience its decline and fall. This must be the natural course of events— the inevitable consequence of spreading knowledge, whether a parliamentary inquiry existed or not. The inquiry itself is a consequence of this tide in the stream of time—a tide which no human ingenuity can arrest, or long impede.

MEDICAL REFORM is now taken out of the hands of angry disputants, and of zealous or interested declaimers, and is in a fair way for a liberal adjustment. As far as we can discern, the investigation has gone almost entirely in favour of REFORM. The STATIONARIES, or advocates of things as they are, have made but a very sorry figure in the examinations before the committee. If not a single individual of the "MOUVEMENT" phalanx had been interrogated, the stationaries would have ruined their own cause before any dispassionate tribunal. They broke down in almost every instance. They either admitted the existence of evils and abuses, or endeavoured to veil them with such a flimsy and gossamer covering, that their deformities became more glaring than ever. It would be premature and improper for us to remark on the evidence which has been adduced before the committee. That evidence will form a volume, which we shall analyze with care and comment upon with freedom. We shall probably be able to furnish our readers with much useful and wholesome food for reflection from the parliamentary report.

If we may judge from the tendency of the questions and the nature of the replies, the following will hold a distinguished place in the catalogue of reforms.

*First,* The establishment of one central board, council, faculty, academy, or whatever else it may be called, for the purpose of regulating the whole of the profession throughout the three kingdoms.

*Secondly,* Uniformity of education, throughout the three kingdoms, for each grade of the profession.

*Thirdly,* It seems probable that there will be only two grades in medicine —a higher and a lower, with a proportionate rate of education and acquirements—and without any other impediment to the passing from the lower to the higher grade, than compliance with the terms prescribed for that higher grade.

*Fourthly,* We think the apprenticeship to the inferior grade will be greatly abbreviated, if not abolished.

*Fifthly,* The legality of charging for medical attendance will be established beyond cavil or doubt, though it is probable that the practitioner will be left the option of claiming his remuneration through the medium of medicines, of skill, or of both conjoined, according to circumstances.

*Sixthly,* We apprehend that a far greater amount of preliminary education will be demanded than now obtains, as well as a much longer period of professional study. This will throw back a little the usual age of commencing practice—a consummation devoutly to be wished.

*Seventhly,* What the amount of power and privilege to be left with the present colleges of physicians and surgeons may be, we cannot conjecture; but we apprehend that the college of apothecaries, in Bridge-street, stands little chance of holding the scales of fate over the general practitioner much longer. Their dominion will probably be transferred to another class of subjects, very useful in their way, but not ranking so high in the science of medicine as of *medicines*.

*Lastly,* It is not improbable that considerable modifications will be made in the management of public hospitals, and in the mode of appointing medical officers, though we doubt whether Parliament can interfere much with institutions that are chiefly, often wholly, supported by private and voluntary subscriptions.

We beg to state that the above are mere conjectures, founded, as we said before, on the tendency of the queries and the nature of the replies in the House of Commons. The alterations and reforms which are here anticipated are those which we *wish,* and, therefore, we may be very much deceived by our own too sanguine hopes. In this case we shall be grieved as well as disappointed. Another year will solve the mystery.

